# Evaluating the reliability of hair analysis in monitoring the compliance of ADHD patients under treatment with Lisdexamphetamine

**DOI:** 10.1371/journal.pone.0248747

**Published:** 2021-03-30

**Authors:** Marianne Haedener, Wolfgang Weinmann, Dominique Eich, Michael Liebrenz, Thomas Wuethrich, Anna Buadze

**Affiliations:** 1 Department of Forensic Toxicology and Chemistry, Institute of Forensic Medicine, University of Bern, Bern, Switzerland; 2 Department of Psychiatry, Psychotherapy and Psychosomatics, Psychiatric Hospital, University of Zurich, Zurich, Switzerland; 3 Department of Forensic Psychiatry, Institute of Forensic Medicine, University of Bern, Bern, Switzerland; Universita degli Studi del Molise, ITALY

## Abstract

Considering the high clinical and forensic relevance of pharmaco-adherence during lisdexamphetamine (LDX) treatment for attention-deficit/hyperactivity disorder (ADHD), the aim here was to evaluate hair analysis as a tool for monitoring compliance in patients currently undergoing long term treatment with LDX, by detecting possible interruptions of medication intake or changes in dosage. For this purpose, a total of 24 patients from an outpatient clinic for ADHD were recruited. Hair and urine samples were taken after three consecutive therapy sessions over a 7-month period and analyzed for amphetamine (AMP) enantiomers and other drugs, using chiral and achiral liquid chromatography-tandem mass spectrometry (LC-MS/MS). Participants also provided information on the condition of their hair, the consumption of illegal psychotropic substances and the regularity of taking LDX. Two participants withdrew from the study early. Urine analyses were positive for D-AMP in all urine samples and therapy sessions, except in two patients who did not take LDX on a daily basis. D-AMP was detected in all hair samples; however, no correlation was found between prescribed dose/day and D-AMP concentrations in proximal hair segments. Qualitative interpretation of hair analysis showed that 18 of the 22 study completers were compliant concerning the intake of LDX without additional consumption of illegal D,L-AMP. Analysis of urine taken during the therapy sessions showed no correlation between D-AMP concentrations and prescribed dosage, with or without normalization for creatinine. In conclusion, chiral LC-MS/MS hair analysis might represent a non-invasive way to confirm LDX use within the approximate period covered by the hair segment tested, but it does not allow for quantitative therapeutic drug monitoring because of interindividual variability of concentrations in hair. Drug concentrations in hair at different stages of long-term treatment should thus be interpreted with caution by clinicians and forensic experts alike when making assessments of treatment adherence.

## Introduction

Attention-deficit/hyperactivity disorder (ADHD) is a common neurobiological disorder predominantly manifesting itself in childhood and persisting into adulthood in an estimated two-thirds of cases [1]. Despite the evidence supporting stimulant treatment and the knowledge of worse outcomes across domains without treatment, many patients remain skeptical of long-term medication use, leading to concerns about adherence. Adherence is commonly defined as a combination of persistence (i.e., whether an individual continues to refill prescriptions in line with the recommended duration of therapy) and compliance (whether an individual actually takes doses according to the issued prescription) [2, 3]. In a literature review, Adler and Nierenberg found that among children and adults, 13% to 64% discontinued their treatment [4]. Understanding the reasons for undertreatment as well as evaluating pharmacological treatment adherence is thus of high clinical, and, as will be seen later, forensic relevance. Stimulants like methylphenidate (MPH) and amphetamine (AMP) are the most commonly prescribed pharmacological treatments for ADHD [5]. In 2007, another pharmaceutical, lisdexamphetamine (LDX) (Vyvanse^®^), became available for treatment, starting in the United States. LDX is an inactive prodrug of D-AMP in which D-AMP is bound by an amide bond to the amino acid L-lysine. Because of the slow and continuous release of D-AMP, the potential for abuse is believed to be lower than that for the immediately active D-AMP and 3:1 D:L-AMP compounds [6–8]. In Switzerland LDX (Elvanse^®^) is approved as a treatment for ADHD in children from 6 years of age, adolescents and—since 2014—also for adults up to 55 years of age who show insufficient response to MPH treatment, thus serving as a second-line treatment [9].

As with all chronic disorders, adherence to treatment for ADHD appears to be a key condition for a successful clinical outcome, i.e., the improvement of cognitive/social functioning and quality of life. In addition, the question of pharmaco-adherence can also arise in a forensic context: for example, in social security law the question of receiving benefits, and, in criminal law, the legal prognosis may be tied to adherence to treatment for ADHD [10]. Indirect methods for evaluating persistence, such as counting tablets, monitoring the filling of prescriptions, self-report diaries or reports from relatives are subject to uncertainty [11, 12]. In contrast, measuring the concentration of the active substance or its metabolites in blood, urine or saliva allows for a more direct evaluation of compliance [13]. However, these measurements usually only provide an indication of compliance, depending on the drug’s pharmacological half-life over several hours to days before the sample was taken, resulting in a “snapshot” rather than a long-term evaluation. By contrast, hair analysis allows for more complete documentation over an extended time period, depending on hair length.

Hair growth rate on the head averages 1 cm per month. It takes about 10–14 days, until the newly grown hair reaches the surface of the scalp and can then be cut; thus, in theory, a 5–6 cm sample would permit the evaluation of medication compliance over about 6 months [14]. The same would be true for 2 cm proximal hair segments taken at three time points with intervals of two months. Plasma and urine concentration of AMP after administration of one or several doses of LDX have been studied in previous pharmacokinetic studies [15–19]. The concentration of AMP in saliva after a single dose of LDX has also been documented [20]. Hair analysis was previously applied to compliance monitoring by Binz et al. after low dose application of 30 to 80 mg/day for 9 patients in long term therapy with LDX, and high inter-individual variations in D-AMP concentrations were found [21]. Prior to introducing chiral AMP testing to evaluate compliance and long-term therapy adherence in LDX-patients, it is therefore paramount to gather reliable individual baseline values of AMP-concentration in hair after regular intake of LDX. Changes in compliance with LDX pharmacotherapy might then be detectable during long-term treatment over several months by changes in D-AMP concentrations in proximal hair segments taken at different time-points.

The primary aim of this study was therefore to evaluate hair analysis as a means of monitoring compliance in patients currently undergoing long-term treatment with Elvanse^®^, i.e. to detect possible interruptions of medication intake or changes in dosage. It was hoped that chiral AMP-testing could be used in the future to evaluate compliance and that, by integrating data on persistence, a more robust understanding of adherence among ADHD-patients taking LDX could be developed. Since a correlation between LDX drug dosage and its concentration in hair was recently suggested, we furthermore aimed to gather more data on this subject in a larger cohort, reflecting clinical reality [21].

The results of this project should not only be of potentially great importance to clinicians evaluating adherence to pharmacological treatment interventions in cases of perceived treatment failure, but also help patients to prove treatment compliance in various forensic contexts, such as for social insurance agencies as part of their obligation to cooperate if receiving a disability pension. The study is also designed to help establish forensic certainty between members of the police force and patients receiving LDX during traffic checks, since it should make it possible to differentiate between legal and illegal AMP consumption.

## Materials and methods

### Compliance with ethical standards

This study was approved by the Cantonal Ethics Committees of Zurich and Bern (project ID 2017–00783). Written informed consent was obtained from all participants. Participants received no compensation for their participation. All procedures performed were in accordance with the ethical standards of the institutional research committee and the 1964 Helsinki declaration and its later amendments or comparable ethical standards.

### Study participants

Individuals were recruited among patients undergoing treatment at the ADHD outpatient clinic of the Psychiatric University Hospital Zurich. Recruitment took place during the regular therapy sessions as well as by phone and postal mail. Inclusion criteria were clinically diagnosed ADHD, treatment with Elvanse^®^ for at least 1 month, age ≥ 18 years, and head-hair length ≥ 1 cm. Exclusion criteria included insufficient knowledge of German and lack of intellectual understanding of the project. Treatment discontinuation with Elvanse^®^, revocation of consent, and impairment of the patient-psychiatrist-relationship (subjective feeling of the patient and/or the treating psychiatrist) were criteria for an early termination of the study.

### Study design and sample collection

The study was carried out within the framework of the regular treatment at the ADHD outpatient clinic of the Psychiatric University Hospital Zurich, which includes bimonthly to quarterly therapy sessions. Urine and hair samples were obtained during three consecutive therapy sessions. Urine samples were collected in 3.5 mL tubes containing 4 mg of sodium azide as a bacteriostatic preservative (Greiner Bio-One, Kremsmünster, Austria) and were stored at -20°C until analysis. Collection of urine specimens did not occur under observation. Sample adulteration (e.g. dilution, chemical manipulation, substitution) seemed unlikely since urine samples were provided voluntarily and the analytical findings had no consequences for the participants. Locks of hair were cut from the posterior vertex region of the patients’ heads, close to the scalp. The samples were wrapped in aluminum foil and stored at room temperature until analysis. Personal and persistence data and was obtained from the patients’ medical records and by oral questioning during the therapy sessions. If patients did not show up for one or more months after their scheduled appointment and/or if they did not renew their prescription, it was considered as treatment discontinuation, which is in line with previous research on treatment adherence [22, 23].

Samples and data were anonymized and sent to the Institute of Forensic Medicine at the University of Bern for analysis. D- and L-AMP concentrations in urine and hair were determined by a chiral liquid chromatography-tandem mass spectrometry (LC-MS/MS) method [24]. Since methamphetamine (MAMP) is metabolized to AMP [25], positive AMP findings in biological specimens can be the result of MAMP consumption. For this reason, hair samples were also analyzed by a well-established LC-MS/MS method for the achiral determination of MAMP and other basic drugs. Findings that might affect the trust between patients and psychiatrists were not reported to the treating psychiatrist and are not presented in this paper (i.e. indications of the abuse of illegal drugs other than that self-reported by the patient).

### Chemicals and reagents

Standards and deuterated internal standards were purchased from Cerilliant (Round Rock, TX, USA). Working solutions for preparing calibration samples for urine and hair analysis were obtained via serial dilution of the standard solution in acetonitrile. Acetonitrile (99.9%, HPLC gradient grade) was acquired from Acros Organics (Chemie Brunschwig, Basel, Switzerland). Ammonium hydroxide solution (25%), methanol, dichloromethane (all EMSURE^®^), and acetone (LiChrosolv^®^) were obtained from Merck (Grogg Chemie, Stettlen, Switzerland). Formic acid solution (puriss p.a., 50% in water) and ammonium formate (LiChropur^®^) were from Sigma-Aldrich (Buchs, Switzerland). Ultrapure water was generated in-house with a Direct-Q water purification system from Millipore (Zug, Switzerland). Drug-free urine and hair samples were donated by employees of the authors’ institute. Urine creatinine concentrations were determined spectrophotometrically on an AU480 analyzer (Beckman Coulter, Nyon, Switzerland) using the Jaffe method [26].

### Preparation of urine samples

Preparation of urine samples for chiral LC-MS/MS analysis was carried out according to a previously published procedure [27]. Briefly, 75 μL of urine sample were thoroughly mixed with 15 μL of internal standard solution (10 mg/L D,L-AMP-*d*_5_) and 1.41 mL of water. The racemic D,L-AMP-*d*_5_ consists of a 1:1 ratio of D-AMP-*d*_5_ and L-AMP-*d*_5_ and is baseline separated by chiral analysis [28]. Calibration samples were prepared by spiking 5 μL of standard working solution into 70 μL of blank urine. Calibration levels were 0.05, 0.1, 0.5, 1, 5, 10, and 25 mg/L per AMP enantiomer, with the lowest calibrator being the lower limit of quantification (LLOQ).

### Preparation of hair samples

In general, the proximal 0–2 cm segment of each collected hair-lock was used for analysis. To remove external contaminations, the hair segments were placed in glass tubes and shaken for 4 min with water, followed by subsequent washing steps with acetone and dichloromethane (with 4 mL each). After drying at room temperature, the proximal hair sample segments were cut with scissors into small pieces and aliquots of 30 mg were weighed into polypropylene tubes. Calibration samples were prepared by spiking 30 μL of standard working solution into 30 mg of finely cut blank hair. For extraction, 10 μL of internal standard solution—containing racemic D,L-AMP-*d*_5_ besides deuterated analogues of other drugs (see below for ’achiral LC-MS/MS analysis’) including MAMP—and 1 mL of methanol were added to the samples. After brief vortex mixing, the samples were incubated in an ultrasonic water bath for 3 h. Subsequently, the samples were centrifuged at 17’000 g and 8 °C for 10 min using a Mikro 220R benchtop centrifuge from Hettich (Bäch, Switzerland). For the chiral LC-MS/MS analysis of AMP, the supernatants were directly transferred to autosampler vials. For the achiral LC-MS/MS analysis of AMP, MAMP and other drugs, the supernatants were diluted 10-fold with a water/acetonitrile/formic acid solution (97.5/2.5/0.1, v/v/v) containing 5 mM ammonium formate. Calibration levels were 50, 125, 250, 1250, and 2500 pg/mg per AMP enantiomer (chiral analysis) and 100, 250, 500, 2500, and 5000 pg/mg for each analyte (achiral analysis), respectively (the lowest calibrator being the LLOQ).

### Achiral LC-MS/MS analysis

Basic drugs and their metabolites (AMP, MAMP, 3,4-methylenedioxymethamphetamine (MDMA), 3,4-methylenedioxyamphetamine (MDA), morphine, 6-acetyl-morphine, codeine, dihydrocodeine, cocaine, benzoylecgonine, cocaethylene, methadone/EDDP, MPH) were determined in hair by a validated achiral LC-MS/MS method. This method comprises chromatographic separation of the analytes on a Kinetex 2.6 μm F5 column (150 x 2.1 mm, Phenomenex, Torrance, CA, USA) using gradient elution with water/acetonitrile containing 0.1% formic acid, followed by tandem-mass spectrometric detection with a 5500 QTRAP^®^ hybrid triple quadrupole/linear ion trap mass spectrometer and a Turbo V ion source (SCIEX, Brugg, Switzerland) operated in positive ESI and SRM mode. Data were acquired and analyzed with Analyst software version 1.6.2 (SCIEX, Brugg, Switzerland). For all analytes that were quantitatively determined, linearity ranges are from 100–5000 pg/mg and the LLOQ is 100 pg/mg. This method has been validated and is used in the accredited laboratory. Further details of this method can be obtained from the authors on request.

### Chiral LC-MS/MS analysis

D- and L-AMP concentrations in urine were determined with a recently developed and validated column-switching LC-MS/MS method [27]. For the analysis of hair samples, the method was slightly adapted and validated according to international guidelines on bioanalytical method validation [29, 30]. Linearity, LLOQ, intra- and inter-batch accuracy and imprecision, selectivity, recovery, matrix effect, process efficiency, carry-over, and re-injection reproducibility were evaluated. Further details of validation data are provided in the S1 File. An aliquot of the diluted urine sample (1 μL) and the hair extract (5 μL), respectively, was directly injected. The liquid chromatography (LC) equipment consisted of an UltiMate 3000 HPLC system (Dionex, Olten, Switzerland) equipped with two binary pumps, an isocratic pump, a thermostatted column compartment equipped with a built-in 6-port switching valve, and a thermostatted plate autosampler. LC was performed with a Gemini 5 μm C18 110 Å trapping column (10 x 2.0 mm) and a Lux 3 μm AMP analytical column (150 x 3.0 mm) combined with a KrudKatcher Ultra in-line filter, all obtained from Phenomenex (CA, USA). Mobile phase A was 0.1 M aqueous ammonia (pH 11), which was freshly prepared on each day of analysis, and mobile phase B was acetonitrile. The autosampler and column oven were maintained at 8 and 30 °C, respectively. A 5 μL aliquot of the methanolic hair extract was loaded onto the achiral C18 trapping column with a 400 μL/min flow of 20% B delivered by binary pump 1. To enhance the loading and trapping step efficiencies, the injection solution was diluted via a T-union with a 200 μL/min flow of mobile phase A delivered by the isocratic pump. After 1 min, the valve was switched and the trapping column was connected to a chiral analytical column in backflush direction. Elution of the enriched and cleaned analytes from the trapping to the analytical column was performed under isocratic conditions with a 500 μL/min flow of 25% B delivered by binary pump 2. During the elution, the flows of binary pump 1 and the isocratic pump were reduced to 20 μL/min since they were diverted into the waste. After 13 min, the valve was switched back to the loading position and the flow rates of the isocratic and binary pump 1 were increased to their initial values. Total run time was 16 min.

Mass spectrometric data were collected in positive electrospray ionization (ESI) and selected reaction monitoring (SRM) mode with a 5500 QTRAP^®^ hybrid triple quadrupole/linear ion trap mass spectrometer. Optimized SRM settings were determined by direct infusion of D,L-AMP and D,/L-AMP-d_5_ via syringe pump and are specified in Table 1.

**Table 1:** Optimized tandem mass spectrometric parameters for the analysis of AMP enantiomers in hair.

Analyte	Q1 mass (m/z)	Q3 mass (m/z)^a^	Dwell time (ms)	DP (V)	EP (V)	CE (eV)	CXP (V)
L-AMP	136.1	**91.0**	150	45	10	26	8
	136.1	119.0	150	45	10	11	8
D-AMP	136.1	**91.0**	150	45	10	26	8
	136.1	119.0	150	45	10	11	8
AMP-*d*_5_	141.1	**93.0**	150	45	10	26	8
Optimized source settings							
Ion spray voltage	3.5 kV						
Source temperature	600 °C						
Curtain gas	40^b^						
Collision gas	Medium						
Gas 1	40^b^						
Gas 2	60^b^						

Q1 quadrupole 1, Q3 quadrupole 3, DP declustering potential, EP entrance potential, CE collision energy, CXP cell exit potential.

^a^Bold: m/z values of ions used for quantification.

^b^ Arbitrary units for the gas settings.

For data analysis, peak area ratios of analyte to internal standard were calculated for each analyte concentration (*x*) and linear least-squares regression with a 1/*x* weighting factor was employed to construct daily calibration curves. Creatinine-normalized urinary AMP concentrations (mg/L) were calculated by dividing AMP concentration (mg/L) by creatinine concentration (mg/dL) and multiplying by a creatinine reference concentration of 100 mg/dL [31]. GraphPad Prism 6.05 software for Windows (GraphPad Software, La Jolla, CA, USA) and Microsoft Excel 2010 were used for data analysis and visualization.

**Fig 1:**
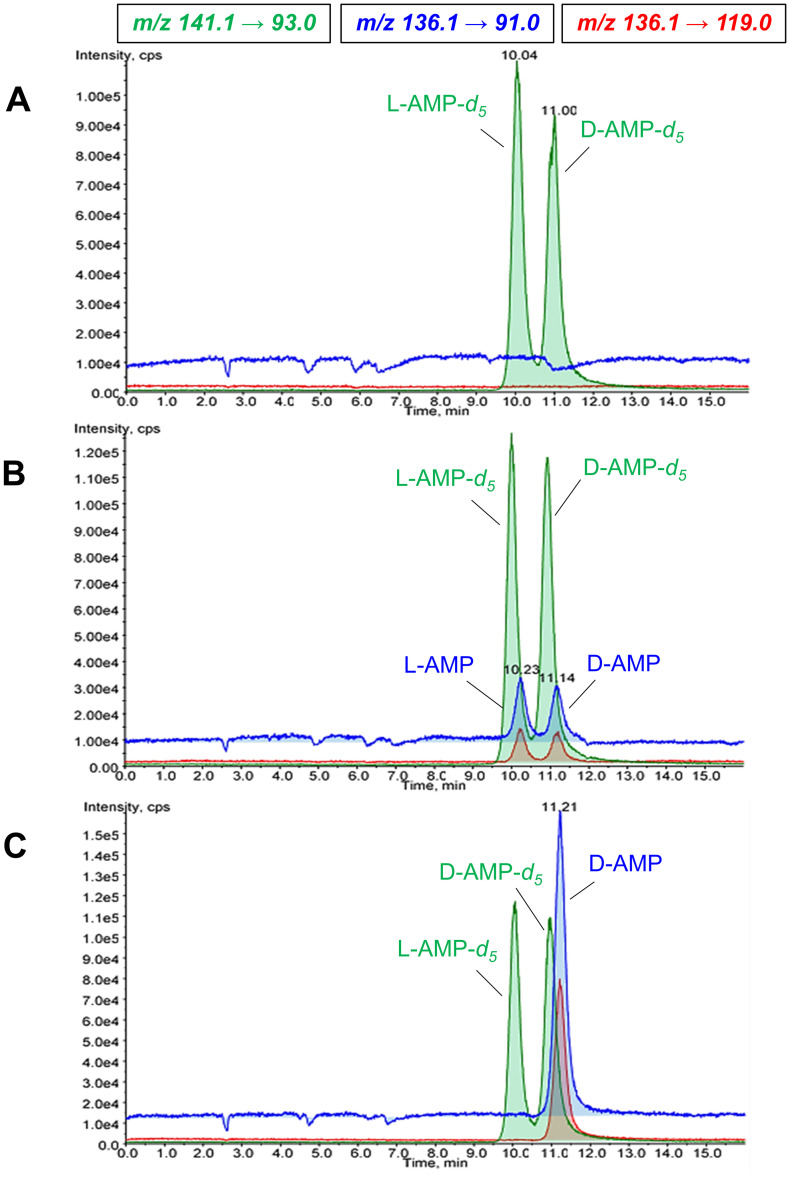
SRM ion chromatograms of (A) blank hair, (B) analytes at the lower limit of quantification (50 pg/mg per AMP enantiomer), and (C) an authentic hair sample from a patient treated with Elvanse^®^. Blue: AMP SRM1 (m/z 136.1 → 91.0); Red: AMP SRM2 (m/z 136.1 → 119.0); Green: AMP-d5 SRM1 (m/z 141.0 → 93.0). Peaks are annotated with retention times: Pane A: 10.04 min: L-AMP-d5; 11.00 min: D-AMP-d5. Pane B: 10.23 min: L-AMP; 11.14 min: D-AMP. Pane C: 11.21 min: D-AMP.

Linear ranges in urine and hair were 0.05 to 25 mg/L and 50–2500 pg/mg, respectively, for both AMP enantiomers. LLOQs for urine and hair were 0.05 mg/L and 50 pg/mg, respectively. Chromatograms of chiral hair analysis are depicted in Fig 1.

### Statistical data evaluation

For testing data sets for correlation between daily Elvanse^®^ dose and D-AMP hair concentrations Microsoft Excel 2010 was used.

## Results

### Participants

Seven men and 17 women participated in the study. Of these, 23 were of European descent and one was Asian. Participants’ characteristics are detailed in Table 2. Participants varied in age (24–59 years) and in body mass index (> 30.0, obese: *n* = 2; 25.0–29.9, overweight: *n* = 4; 18.5–24.9, normal: *n* = 18). Six individuals received Elvanse^®^ in combination with MPH (*n* = 5) or D-AMP sulfate (*n* = 1, 5–10 mg/day). Several participants received concomitant medication for other mental health conditions. Seven participants self-reported use of illegal drugs (cannabis (n = 4), cocaine (n = 1), AMP (n = 2), MDMA (“ecstasy”, n = 1)). Two participants withdrew from the study early (due to treatment discontinuation with Elvanse^®^ and for personal reasons, respectively).

**Table 2:** Participants’ characteristics, diagnosis, treatment, and self-reported drug abuse.

Participant	Ethnicity^a^	Sex	Age^b^ (years)	Body mass index (kg/m^2^)	ICD-10 diagnosis^c^	Elvanse^®^ use (months)^d^	Elvanse^®^ dose (mg/day)^e^	Concomitant medication	Self-reported drug abuse
1	E	m	26	33.7	F90.0 F32.1	3	50	escitalopram	nicotine
2	E	f	55	27.5	F90.0 F32.1	16	50	sertraline	-
3	E	f	27	23.0	F90.0	20	60	-	-
4	E	m	26	23.4	F90.0	1	40 / 90	quetiapine / duloxetine	cannabis
5	E	m	37	21.9	F90.0	20	320	-	-
6	E	m	49	21.6	F90.0 F10.2 F14	32	70	-	cocaine, nicotine
7	E	m	24	20.8	F90.0 F17.2	16	30 / 50	-	AMP, nicotine, cannabis
8	E	f	27	20.7	F90.0	32	40	-	-
9	E	f	35	25.7	F90.0 F17.2	20	50	-	nicotine
10	E	f	46	20.4	F90.0	14	30	-	-
11	E	m	34	23.4	F90.0	27	30	-	-
12	E	f	52	41.5	F90.0 F50.9	33	30	MPH	-
13	E	f	45	19.6	F90.0	34	30–50^f^	MPH	MDMA
14	A	f	45	20.2	F90.0	13	30 / 40 / 70	venlafaxine	-
15	E	f	24	20.8	F90.0	6	60	-	cannabis
16	E	f	38	26.1	F90.0 E06.3 L93	17	50	MPH	-
17	E	f	42	22.0	F90.0 F33	3	20 or 30^g^	sertraline	nicotine
18	E	f	43	21.0	F90.0 F50.2	1	30 / 60	fluoxetine	-
19	E	m	41	29.4	F90.0 F12.1	13	100	-	AMP
20	E	f	59	23.5	F 90.0	26	50	MPH	-
21	E	f	34	19.0	F90.0 F12.2	24	100	D-AMP sulfate (5–10 mg/day)	cannabis, nicotine
22	E	f	34	20.3	F90.0	37	80	-	-
23	E	f	45	22.0	F90.0	2	40	MPH	-
24	E	f	48	23.5	F90.0 M33.1	38	80	human immune globuline	-
Completers (1–22)									
Median			37.5	21.9		18.5			
Mean			38.3	23.9		18.5			
SD			10.3	5.3		11.3			
All (1–24)									
Median			39.5	22.0		18.5			
Mean			39.0	23.8		18.7			
SD			10.1	5.1		12.0			

^a^E, European; A, Asian.

^b^ Age at the time of the first sample collection.

^c^ ICD-10, International Classification of Diseases, 10^th^ revision.

^d^ Duration of Elvanse^®^ treatment prior to the first sample collection.

^e^ Slashes indicate that the prescribed Elvanse^®^ dose changed during the study.

^f^ Participant takes variable doses of Elvanse^®^, but not on a daily basis.

^g^ Participant takes alternating doses of Elvanse^®^.

### Urine analysis

Creatinine-normalized urinary D-AMP concentrations measured in study completers are presented in Table 3. Creatinine is a by-product of muscle metabolism. It is normally excreted into urine at a constant rate and its level in the urine reflects the urine concentration. Normalizing urinary drug concentrations to a constant creatinine concentration is useful to account for intra- and interpersonal differences in urine dilution (e.g. caused by excessive or reduced fluid intake) [31, 32]. All but participants 13 and 15 tested positive for D-AMP at all three time points. The absence of D-AMP in the urine samples of participant 13 is not too surprising since this participant reported to the treating psychiatrist that she hadn’t been taking Elvanse^®^ on a daily basis. The absence of D-AMP in the second and third urine sample of participant 15 may indicate non-compliance with the prescribed Elvanse^®^ regimen, but could also be due to inappropriate timing of urine collection relative to Elvanse^®^ intake. In our study, time intervals between last Elvanse^®^ intake and urine collection in our study were not controlled and ranged approximately from 0.5 h– 30 h. D-AMP concentrations, even after creatinine-normalization, displayed high intra- and intersubject variability. No correlation between prescribed Elvanse^®^ dose and D-AMP concentration was observed. Participants 6 and 7 tested positive for L-AMP at one and two time points, respectively. Participant 7 self-reported abuse of illegal racemic AMP. Measured L-AMP concentrations were approximately 10–100 times lower than D-AMP concentrations.

**Table 3:** Results of the urine analysis. D- and L-AMP concentrations are normalized to a creatinine reference concentration of 100 mg/dL.

Participant	Sampling time (months)^a^	Elvanse^®^ (mg/day)^b^	D-AMP (mg/L)	L-AMP (mg/L)
1	0	50	7.25	n.d.
	2.2	50	1.52	n.d.
	4.1	50	3.11	n.d.
2	0	50	12.88	n.d.
	1.6	50	7.97	n.d.
	4.6	50	2.51	n.d.
3	0	60	21.49	n.d.
	2.8	60	10.60	n.d.
	4.6	60	8.78	n.d.
4	0	40	7.56	n.d.
	2.5	90	6.49	n.d.
	5.5	90	4.83	n.d.
5	0	320	12.07	n.d.
	2.3	320	8.40	n.d.
	4.4	320	5.30	n.d.
6	0	70	5.24	0.43
	1.8	70	5.46	n.d.
	6.6	70	3.96	n.d.
7	0	30	6.34	0.13
	1.8	50	11.21	n.d.
	4.7	50	6.64	0.06
8	0	40	8.58	n.d.
	5.2	40	1.80	n.d.
	7.6	40	3.11	n.d.
9	0	50	0.17	n.d.
	2.0	50	1.88	n.d.
	4.7	50	1.08	n.d.
10	0	30	2.32	n.d.
	2.1	30	3.46	n.d.
	4.1	30	3.82	n.d.
11	0	30	2.41	n.d.
	3.6	30	2.90	n.d.
	6.0	30	1.30	n.d.
12	0	30	3.98	n.d.
	2.4	30	2.83	n.d.
	3.9	30	0.06	n.d.
13	0	30–50^c^	- ^f^	- ^f^
	1.9	30–50^c^	n.d.	n.d.
	4.5	30–50^c^	n.d.	n.d.
14	0	30	10.75	n.d.
	4.7	40	13.48	n.d.
	7.3	70	16.83	n.d.
15	0	60	15.21	n.d.
	3.6	60	n.d.	n.d.
	5.6	60	n.d.	n.d.
16	0	50	0.86	n.d.
	3.8	50	0.84	n.d.
	5.5	50	6.93	n.d.
17	0	20 / 30^d^	7.72	n.d.
	3.4	20 / 30^d^	4.79	n.d.
	5.5	20 / 30^d^	3.28	n.d.
18	0	30	12.63	n.d.
	1.7	60	2.80	n.d.
	4.8	60	3.68	n.d.
19	0	100	6.39	n.d.
	1.8	100	5.25	n.d.
	4.8	100	0.20	n.d.
20	0	50	3.54	n.d.
	2.3	50	10.04	n.d.
	4.1	50	3.09	n.d.
21	0	100^e^	17.53	n.d.
	2.0	100^e^	13.78	n.d.
	4.4	100^e^	14.37	n.d.
22	0	80	1.05	n.d.
	1.6	80	0.39	n.d.
	2.6	80	0.40	n.d.

*n*.*d*. not detected

^a^ Time of sample collection after the beginning of the study.

^b^ Prescribed dose of Elvanse^®^ during the month before sample collection.

^c^ Participant takes variable doses of Elvanse^®^, but not on a daily basis.

^d^ Participant takes alternating doses of Elvanse^®^.

^e^ Participant receives Elvanse^®^ in combination with D-AMP sulfate (5–10 mg/day).

^f^ No urine sample was collected.

### Hair analysis

Example chromatograms of chiral analysis for D- and L-AMP are shown in Fig 1, showing that baseline separation is achieved for the AMP-enantiomers. Results of the chiral LC-MS/MS analysis of hair samples are given in Table 4. All study completers showed D-AMP positive hair segments. Participant 13, however, who did not take Elvanse^®^ on a daily basis, only tested positive for D-AMP during the third time period (approx. 2.5–4.5 months after the beginning of the study). L-AMP could be detected in the hair samples of four participants (No. 7, 13, 15, and 19). For participants 7 and 19, who had self-reported abuse of illegal racemic AMP, L-AMP concentrations were clearly lower than D-AMP concentrations in all analyzed hair segments (1.7–6.8 times), suggesting only occasional co-consumption of street AMP. Subjects 13 and 15, however, showed D/L ratios of 0.8–1.4, which strongly indicates that they primarily consumed racemic street AMP rather than the prescribed dose of Elvanse^®^.

**Table 4:** Results of the hair analysis.

Participant	Hair color	Sampling time (months)^a^	Segment (cm)	Elvanse^®^ (mg/day)^b^	D-AMP (pg/mg)	L-AMP (pg/mg)	MPH positive
1	blonde	0	2	50	2000	n.d.	
		2.2	2	50	1218	n.d.	
		4.1	2	50	1193	n.d.	
2	dark blonde	0	2	50	355	n.d.	
		1.6	2	50	182	n.d.	
		4.6	2	50	347	n.d.	
3	black*	0	14	60	115	n.d.	x
		2.8	2	60	120	n.d.	x
		4.6	2	60	< 50	n.d.	x
4	brown	0	1	40	96	n.d.	x
		2.5	2	40 / 90	586	n.d.	
		5.5	2	90	422	n.d.	
5	brown*	0	2	320	360	n.d.	
		2.3	2	320	232	n.d.	
		4.4	2	320	223	n.d.	x
6	brown-grey	0	2	70	95	n.d.	x
		1.8	2	70	134	n.d.	x
		6.6	2	70	118	n.d.	x
7	brown	0	2	30	384	121	
		1.8	2	30 / 50	292	173	
		4.7	2	50	341	< 50	
8	dark blonde*	0	2	40	716	n.d.	
		5.2	2	40	797	n.d.	
		7.6	2	40	923	n.d.	
9	dark blonde	0	2	50	86	n.d.	
		2.0	2	50	103	n.d.	
		4.7	2	50	71	n.d.	
10	grey*	0	2	30	111	n.d.	
		2.1	2	30	235	n.d.	
		4.1	2	30	60	n.d.	
11	black	0	2	30	222	n.d.	
		3.6	3	30	72	n.d.	
		6.0	2	30	277	n.d.	
12	dark brown	0	2	30	236	n.d.	x
		2.4	2	30	< 50	n.d.	x
		3.9	2	30	108	n.d.	x
13	dark blonde*	0	2	30–50^c^	n.d.	n.d.	
		1.9	2	30–50^c^	n.d.	n.d.	
		4.5	2	30–50^c^	142	124	x
14	dark brown	0	2	30	321	n.d.	
		4.7	2	40	156	n.d.	
		7.3	2	70	393	n.d.	
15	red*	0	2	60	1538	1078	
		3.6	2	60	1076	1272	
		5.6	2	60	3209	3763	
16	dark blonde*	0	2	50	139	n.d.	x
		3.8	2	50	67	n.d.	x
		5.5	2	50	610	n.d.	x
17	black*	0	2	20 / 30^d^	956	n.d.	x
		3.4	2	20 / 30^d^	1711	n.d.	
		5.5	2	20 / 30^d^	1003	n.d.	
18	brown*	0	2	0 / 30	518	n.d.	x
		1.7	2	30 / 60	1179	n.d.	x
		4.8	2	60	2866	n.d.	x
19	brown-grey	0	2	100	303	n.d.	
		1.8	2	100	214	<50	
		4.8	2	100	393	n.d.	
20	brown	0	2	50	468	n.d.	
		2.3	2	50	524	n.d.	x
		4.1	2	50	2014	n.d.	x
21	brown	0	2	100^e^	291	n.d.	x
		2.0	2	100^e^	355	n.d.	x
		4.4	2	100^e^	735	n.d.	
22	brown	0	2	80	283	n.d.	
		1.6	2	80	177	n.d.	
		2.6	2	80	194	n.d.	

*n*.*d*. not detected

*Cosmetic treatment

^a^ Time of sample collection after the beginning of the study.

^b^ Prescribed dose of Elvanse^®^ during the time period represented by the hair segment. Slashes indicate that the prescribed Elvanse^®^ dose changed during the time period.

^c^ Participant takes variable doses of Elvanse^®^, but not on a daily basis.

^d^ Participant takes alternating doses of Elvanse^®^.

^e^ Participant receives Elvanse^®^ in combination with D-AMP sulfate (5–10 mg/day).

AMP concentrations measured with the chiral LC-MS/MS method were found to be in good agreement with those obtained with our routine LC-MS/MS method for achiral determination of AMP, MAMP, MPH and other basic drugs. For hair samples with total AMP concentrations ≥ 100 pg/mg (LLOQ of the achiral analysis; n = 55), the results of the chiral analysis were within 80.9–122.9% (153.3% for one sample) of the results of the achiral analysis.

MAMP could not be detected in any of the collected hair samples indicating that none of the participants consumed MAMP on a regular basis. Sporadic MAMP consumption cannot be excluded, but the AMP derived from the metabolism of sporadically consumed MAMP would not make a significant contribution to the measured AMP hair levels. The self-reported cocaine use of participant 6 and MDMA use of participant 13 were confirmed through detection of cocaine and its metabolites (benzoylecgonine, ecgonine methyl ester, cocaethylene, norcocaine) and MDMA, in all analyzed hair segments. All participants who were prescribed MPH as well as seven additional participants showed MPH- positive hair segments (limit of detection 2 pg/mg), as displayed in Table 4.

**Fig 2:**
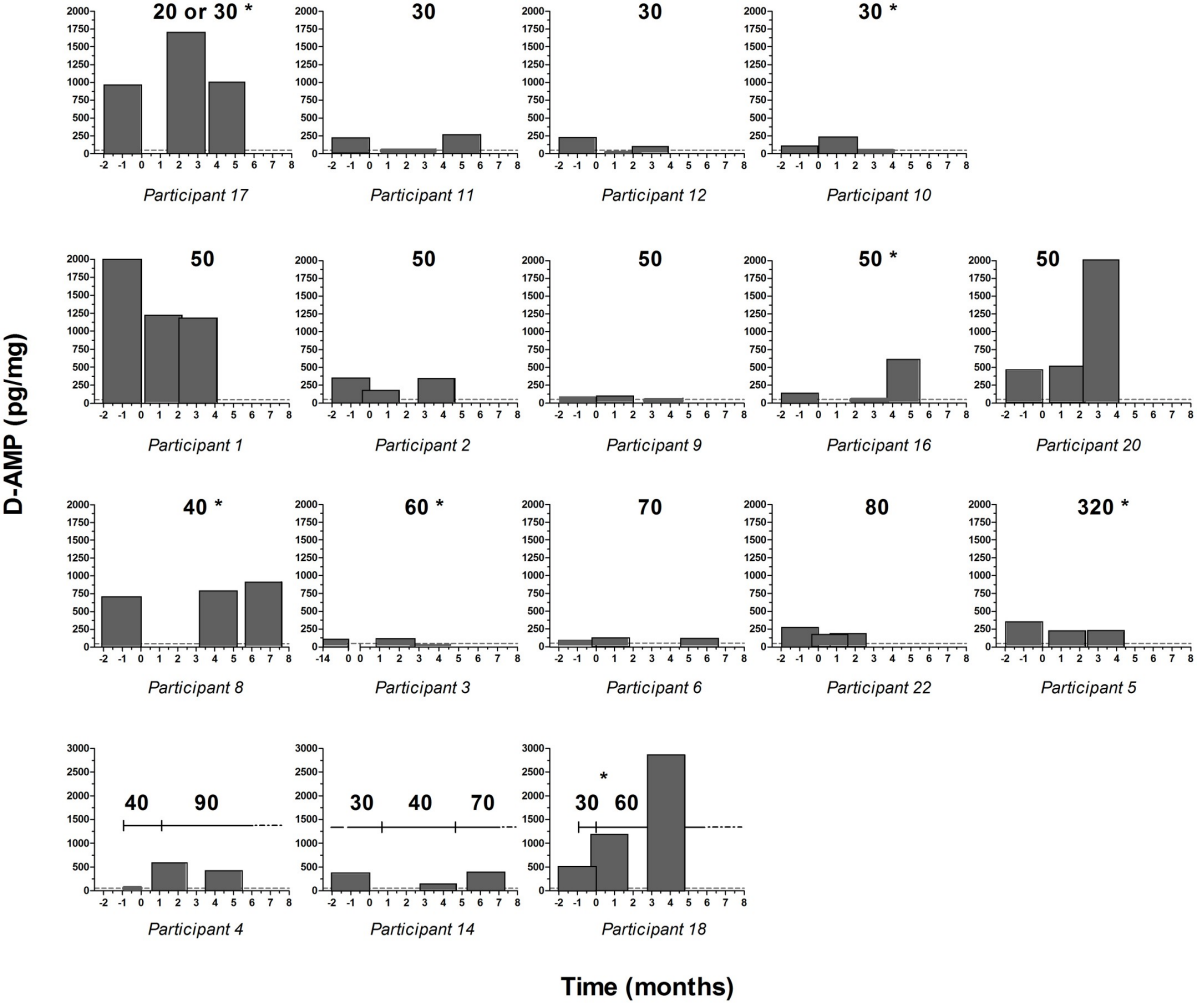
D-AMP concentrations in hair. Bars represent hair segments (assuming an average hair growth of 1 cm/month). Time = 0 corresponds to the time of the first sample collection. Numbers in bold indicate prescribed Elvanse^®^ dose, asterisks indicate cosmetic treatment, and dotted lines indicate the limit of quantification (50 pg/mg).

D-AMP concentrations in hair segments were highly variable within participants and between participants receiving the same Elvanse^®^ dose, as is graphically illustrated in Fig 2. Participants who were found to consume racemic street AMP or received Elvanse^®^ in combination with D-AMP sulfate were not included in Fig 2, since the measured D-AMP concentrations were not solely the result of Elvanse^®^ intake. For participants, who received a constant Elvanse^®^ dose throughout the study, there was a factor of 1.3–9.1 (median 2.2) between the lowest and the highest D-AMP concentration measured in the three hair segments. Three participants underwent dose adjustment during the study. However, only for one of them (Fig 2, pane at the bottom/right, patient 18), were changes in prescribed dose clearly reflected by the observed D-AMP concentration changes in the three hair segments.

No correlation between prescribed Elvanse^®^ dose and D-AMP concentration could be established among participants. Participants who were prescribed 50 mg/day, for example, showed hair D-AMP concentrations from 71 to 2000 pg/mg. Furthermore, participant 5, who was supposed to take 320 mg per day, showed much lower D-AMP concentrations in hair than did some participants who were prescribed lower doses, e.g. subject 17 (20 / 30 mg), subject 8 (40 mg) and subject 1 (50 mg). Even after elimination of those patients, who had chemically treated hair (marked with an asterisk in Table 4), and additional elimination of patient 7, for whom additional D/L-Amp uptake was detected in hair, no correlation was found (Pearson correlation coefficient r = -0.14) between daily Elvanse^®^ dose (in mg) and hair D-Amp concentrations (pg/mg).

## Discussion

Considering the high clinical and forensic relevance of pharmaco-adherence when treating individuals with LDX for the neurodevelopmental disorder ADHD, the primary aim of this study was to evaluate hair analysis for monitoring compliance in patients currently undergoing long term treatment with Elvanse^®^, i.e. and to detect possible interruptions of medication intake or changes in dosage. Since one small previous study reported high inter-individual variations but simultaneously suggested a dose response relationship with a correlation coefficient of 0.64 between concentration in hair and LDX dosage, we furthermore aimed to gain more insight into this finding [21]. Additionally, we obtained data on persistence, by evaluating treatment continuity to obtain a more thorough understanding of adherence among individuals suffering from ADHD and receiving LDX.

We found that individuals undergoing long-term treatment with Elvanse^®^ were positive for D-AMP in all urine samples and therapy sessions, except for two patients who did not take LDX on a daily basis. D-AMP was detected in all hair samples of participants taking prescribed LDX in dosages between 20 and 320 mg on a daily basis, adding further evidence to the feasibility of chiral LC-MS/MS analysis to confirm LDX use within the approximate period covered by the hair segment tested.

However, no correlation was found between D-AMP concentrations and prescribed dosage, either by analysis of urine taken during the therapy sessions as spot urine samples, with or without normalization for creatinine, or by analysis of proximal hair segments. The latter finding contradicts earlier reporting published elsewhere and casts doubt on the current usefulness of chiral LC-MS/MS analysis for monitoring treatment compliance beyond a basic estimation of whether or not the drug has been taken regularly over a long period of time. Our results not only imply that unchanged dosages might lead to a high variance in D-AMP readings but, furthermore, that clinically significant increases in dosage of LDX do not lead to a linear increase in D-AMP concentrations in proximal hair segments.

Our results are based on a sample that corresponds to clinical reality in a number of variables, such as co-morbidities, concurrent medication and substance abuse and also common hair treatment (see Tables 2 and 4). As numerous studies show, up to 80% of individuals with a diagnosis of adult ADHD suffer from a comorbid psychiatric disorder such as harmful use of or dependence on psychotropic substances (SUD), affective disorders and/or anxiety disorders [33–35]. In a clinical setting, SUDs pose a major challenge, both diagnostically and therapeutically. On the one hand those suffering from ADHD have an increased risk for SUD and, on the other, 20–30% of individuals who abuse psychoactive substances also suffer from ADHD [36]. To treat these psychiatric comorbidities, patients with ADHD often receive other medications in addition to stimulant treatment, on either a short- or long-term basis. In our sample this concurrent medication predominantly consisted of selective serotonin reuptake inhibitors (SSRIs) and serotonin and norepinephrine reuptake inhibitors (SNRIs) for individuals with co-occurring depressive symptoms or bulimia nervosa. The use of these drugs for the aforementioned disorders is thereby in line with current treatment recommendations [37–39].

In 5 cases (6 cases including non-completers) a dose of more than 70 mg of Elvanse^®^ was necessary to achieve a significant symptom reduction. These dosages must be seen against the background of LDX being a second line treatment for ADHD in Switzerland. As such, the sample comprised predominantly patients who were MPH non-responders and/or who suffered from MPH side effects. The chronological sequence of a switch from first line MPH to second line LDX also explains why in some cases MPH was found in addition to LDX in hair analysis. Furthermore, it should be mentioned that in the case of participant 21, medication solely with LDX was perceived to be insufficient, resulting in functional impairments at the workplace, especially in the early morning hours and a combination with D-AMP was initiated by the treating psychiatrist. This was done to bridge the gap until LDX had reached peak plasma concentration. Thus, dosages of the medication in this sample are also a reflection of clinical reality, with sometimes high dosages of LDX and MPH as add-ons.

When non-correlation between dose and concentrations found in urine (or hair) are discussed, several issues need to be considered, such as daily dose per kg bodyweight, time of sample collection after dosage, intra- and inter-subject variability in pharmacokinetics and metabolism, including time to reach steady state concentrations, clearance and dilution of the urine.

Controlled studies with urinary excretion data, however, are scarce and detailed urine concentration-time profiles for LDX and D-AMP have not yet been established. To our knowledge, only two studies have documented AMP concentrations in urine following LDX administration. Thevis et al. [19] analyzed urine samples obtained from a patient receiving 30 mg of Elvanse^®^ once daily (corresponding to 17.3 mg of LDX free base) and being at pharmacokinetic steady-state; they reported urinary AMP concentrations of 0.04, 0.11, 0.45, and 1.15 mg/L at 3, 6, 9, and 11 h post-administration. Comiran et al. [20] collected urine samples from six male volunteers 2 h after they had been administered a single oral dose of 70 mg of LDX dimesylate (corresponding to 40.5 mg of LDX free base). Mean urinary AMP concentration was 1.51 mg/L (no creatinine-correction in either study). In a study with D-AMP administration, Poklis et al. [40, 41] determined urinary D-AMP concentrations and also found substantial variability. D-AMP concentrations in six random urine specimens collected over a 3-day period from an adult at steady-state receiving 30 mg of D-AMP daily were found to range from 1.1 to 17.8 mg/L. Healthy male volunteers receiving a single oral dose of 5 mg, 10 mg, or 20 mg of D-AMP displayed a wide range in peak urinary D-AMP concentrations at each dose and concentration ranges that overlapped considerably following the various doses. These findings highlight the difficulty of assessing the ingested dose or time since last dose based upon the drug concentration measured in a single random urine specimen. Since comprehensive studies of urinary elimination are lacking and since urine samples were collected at unknown time points after LDX intake in the present study, it is difficult to assess whether the D-AMP concentrations measured herein support daily compliance with the prescribed Elvanse^®^ regimen or are the result of non-daily intake.

In addition, it must be kept in mind that the CYP2D6 enzyme which mediates aromatic hydroxylation of AMP is known to exhibit significant genetic polymorphism, including marked interethnic variation [42]. However, the lack of correlation between LDX dose and resultant urinary D-AMP concentrations most likely lies in the fact that the time of urine collection in relation to the last LDX dose was not controlled and was certainly variable. Exact time intervals between last LDX intake and urine sampling were not recorded in our study.

For hair analysis, time period of dosage as well as variations in hair growth, hair color, sweat and sebum production, length and thickness of hair, cosmetic hair treatment, hair washing, etc. are important factors influencing drug concentrations. However, hair analysis has enjoyed increasing interest as an alternative or complementary matrix to blood and urine in forensic and clinical drug testing, as it provides a larger window of detection and a long-term history of drug use. Compared to the analysis of blood and urine, hair testing has further advantages, such as a reduced risk of sample adulteration, ease of sample collection, transport and storage, and stability of the incorporated substances [43, 44].

Drug incorporation into hair is recognized to occur via multiple mechanisms and at various times during the hair growth cycle. Proposed mechanisms include passive diffusion from the systemic blood circulation to the actively growing follicle, deposition by diffusion from sebum or sweat into the hair follicle after formation, and external contamination after the hair has emerged from the skin [45].

Incorporation of drugs from the bloodstream depends on the average concentration in the blood over time and thus on the ingested dose. Systemic exposure to D-AMP (maximum plasma concentration [C_max_], area under the plasma concentration-time curve [AUC]) shows low intrasubject and intersubject variability and appears to be proportional to oral LDX dose, indicating that there is no enzyme saturation in the conversion of LDX to D-AMP. Further findings suggest that steady-state D-AMP plasma concentrations are reached after five daily oral doses of LDX, with no evidence of LDX or D-AMP accumulation. The pharmacokinetics of LDX and D-AMP did not differ as a function of gender or age when the LDX dose was normalized by body weight [15–17, 46–49]. With passive diffusion, it would be expected that a correlation between hair concentration and steady-state D-AMP plasma concentration, and thus ingested LDX dose, can be established for compliant patients.

However, physicochemical properties of drugs (melanin affinity, lipophilicity, and basicity) are a more important factor for incorporation from blood into hair than plasma concentrations. It is well documented that hair pigmentation (melanin content) is an important factor influencing the incorporation of basic drugs such as AMP into hair, which may lead to a so-called color bias. Dark-haired persons (higher melanin concentrations) were found to have much higher concentrations of basic drugs than subjects with lighter hair when exposed to the same dose [50–53].

The participants in the present study had different natural hair colors, and nine of them reported using cosmetic hair treatments (mostly dyeing, but also bleaching and thermal straightening) as indicated in Table 3. Cosmetic treatments were found to decrease the concentrations of AMP in hair [54–56]. This as well as the possibility of color bias need to be kept in mind when interpreting the hair results.

Only few comparisons could be drawn between participants having cosmetically untreated hair and receiving the same Elvanse^®^ dose. Blonde-haired participant 1 had higher mean hair D-AMP concentrations than brown-haired participant 20, which contradicts expectations based on the melanin-dependent incorporation of basic drugs. Participants 2 and 9, however, both having dark blonde hair and receiving an Elvanse^®^ dose of 50 mg/day, had lower mean hair D-AMP concentrations than brown-haired participant 20 (294 pg/mg and 87 pg/mg, respectively, versus 1002 pg/mg). Participants 11 and 12, who both received a daily Elvanse^®^ dose of 30 mg and had dark hair (black and dark brown, respectively) had similar D-AMP concentrations in their hair.

When interpreting the hair D-AMP concentrations measured in the present study, the possible contribution of incorporation mechanisms other than diffusion from blood must also be considered (diffusion from sebum/sweat and external contamination). Numerous studies have demonstrated the detection of drugs and their metabolites in sweat/sebum, including AMP and MPH [57]. Individual variability in sweat and sebum secretion could contribute to the observed variability in hair D-AMP concentration in subjects receiving the same Elvanse^®^ dose.

From a clinical perspective adherence (a combination of persistence and compliance) to therapy is an important predictor for treatment outcome and may be problematic for patients with mental health issues in general, but especially for patients with attention deficit hyperactivity disorder due to symptoms such as concentration problems, inattention and disorganization, which might lead to an “unintentional nonadherence” [58]. A more recent review by Adler and Nierenberg reported that nonadherence and medication discontinuity increases when patients are followed for longer periods of time and found mean continuities of usually less than 6 months [4]. It also revealed that most of the studies on this topic address the issue in children but not in adults, despite the fact that ADHD persists well into adulthood. The authors raise the question of whether monitoring pharmacotherapy could influence adherence and if so, in what way. Interestingly, the rates of interruption of medication were lower for long-acting preparations compared to those for immediate release formulations. This result underlines the fact that patients with ADHD do not use the prescribed drugs primarily for recreational purposes, i.e. they do not want to achieve a "flash" although some misuse has been described among diagnosed adolescents taking prescription stimulants [59]. Our findings concerning LDX as the formulation with the longest duration of efficacy among the currently available drugs for ADHD are in alignment with this data. While our study only investigated adherence to LDX and did not directly compare subjects prescribed short-acting ADHD medication, our results indicated that in the LDX group of 24 patients, 18 patients (75%) showed good compliance and persistence with the medication over the monitored time-period, with only two patients withdrawing from the study. These numbers are significantly higher than the numbers reported by Adler and Nierenberg for short-acting preparations, where 13.2% to 64% discontinued their treatment. Moreover, in comparison to other medications used in psychiatry, e.g. antidepressants, we can report good adherence to treatment with LDX in our sample of adults with ADHD. For example, Sawada et al. investigated persistence and compliance with antidepressant treatment among individuals with depression and found that only 44.3% continued antidepressant treatment for 6 months. Furthermore, 63.1% of patients discontinuing their initial antidepressant, did so without consulting their treating physician [60, 61].

From a forensic point of view, the results of the present study have significant implications for the assessment of treatment adherence in a medico-legal context. To exemplify, these implications will be discussed in the areas of criminal law, social law and traffic law in the Swiss juridical system. In criminal law, in particular, release from therapeutic measures according to the Swiss Criminal Code (SCC) is linked to a reduced risk of reoffending [62, 63]. Therapy adherence is considered an important variable during risk assessment [64, 65].

If, as suggested by other authors, results are interpreted quantitatively with a given coefficient linking dose and response by legal authorities, those affected could be suspected of not following treatment protocol with regards to prescribed medication, despite regularly taking a constant dose and being persistent and compliant. A lack of adherence to therapy, on the other hand, has a negative effect on the assessment of the prognosis for relapse and can thus unjustifiably prolong the duration of court mandated treatment and represent an obstacle to successful rehabilitation [66].

Since the prescription of LDX (or other stimulants for that matter) to individuals in detention who suffer from ADHD is an exception, decreasing readings of AMP despite stable doses in the hair, could, if interpreted quantitatively, suggest deviation and misuse of LDX by those individuals, which might lead to dire, albeit unwarranted, consequences [67–70]. Prescriptions for stimulants are often not filled because it is assumed by prison authorities that the substance will be “smuggled” to other inmates; thus such an interpretation has consequences not only for the patients themselves, but possibly also for other detainees suffering from this highly prevalent disorder who are in need for treatment [71–73]. On a related note, it should be added that positive AMP findings in urine and hair may not only be the consequence of the misuse of an illegal substance, but also the consequence of taking another prescribed drug such as famprofazone, a nonsteroidal anti-inflammatory agent (NSAID). If the results of hair analysis are not interpreted carefully, an individual in custody may thus be subject to unjustified sanctions, including confinement [74].

In social law, on the other hand, the distribution of benefits, either in the form of disability pensions or professional reintegration efforts, are also determined by adherence to therapy. There is a so-called “obligation to cooperate” that is designed to minimize the financial consequences for society of sustained disability [75, 76]. Here, too, those affected may suffer considerable disadvantage if, based on forensic experts’ testimony, legal authorities interpret the results of hair-analysis for LDX quantitatively. It is conceivable, for example, that vocational training will not be financed or initiated, as it is assumed that the individual concerned does not show therapy-adherent behavior.

Mobility and possession of a valid driver’s license are prerequisite for employment in many professional constellations, and access to an automobile increases not only the chances of being employed but influences the quality of employment, as measured by earnings [77, 78]. If, in the context of police traffic stops or traffic medical examinations, conclusions about illegal drug use and treatment compliance are drawn without acknowledging the limitations of chiral LC-MS/MS hair analysis, the results can lead to a massive burden for the individual, both professionally and socially [79, 80].

Nevertheless, if the data are carefully interpreted and limitations are openly communicated to legal authorities, chiral hair analysis for D-AMP and L-AMP can represent an instrumental enrichment for both forensic and general psychiatrists, since confirming LDX use within the approximate period covered by the hair segment tested, can be one (further) objective element when assessing therapy adherence.

## Limitations

A major concern of the study participants was that too much hair would be collected and that hair sampling would negatively impact their hair cut and leave visible bald spots on their scalp. Therefore, only one lock of hair was taken at each time point and only as much hair as necessary for the analysis was cut, although the homogeneity from thicker hair-locks was arguably better.

The question of the representativity of a single hair-lock for drug analysis was addressed by studies of Dussy et al. [81] and Meier et al. [82]. Dussy et al. [81] showed that incorporation of substances into head hair is not necessarily uniform and that significant differences in analyte concentrations may be found in hair-locks sampled from the same person covering the same time period, depending on the sampling site on the head. A limitation of the hair analysis here is that, due to the aforementioned objection (visibility of bald spots), only one hair sample was taken and analyzed at each time point.

Furthermore, interpretation of segmental hair analysis results is restricted by experimental as well as biological sources of inaccuracy. Experimental errors can occur during fixing and cutting a hair-lock from the scalp prior to segmentation. In routine samples, this can lead to a mutual shift of single hair-strands by up to 5 mm corresponding to a time error of about two weeks. However, great care was taken to avoid such errors in the present study.

## Conclusions

In light of the negative consequences associated with intentional or unintentional non adherence to stimulant treatment for individuals with ADHD [58, 59], chiral LC-MS/MS hair analysis for patients receiving LDX might be a practical non-invasive way to confirm LDX use within the approximate period covered by the hair segment tested. However, currently hair analysis cannot be used in "compliance monitoring" where "compliance" means appraising whether an individual is taking doses according to the prescription issued. It does not provide information on dose or frequency of LDX use. This might be due to the interindividual variability of concentrations in hair produced by factors such as hair pigmentation and cosmetic hair treatments, but also diffusion from sweat and external contamination. Hair drug concentrations at different stages of a long-term treatment should thus be interpreted with caution by forensic and legal experts alike when making assessments of treatment adherence, since unchanged dosages of LDX might not only lead to a high variance in hair drug concentration, but also to conditions where clinically significant increases in dosage of LDX might not be reflected at all.

Forensic experts advising courts and/or other legal entities should furthermore keep in mind that AMP in urine or hair may not only be the result of methamphetamine abuse, but also a consequence of the intake of prescription or over the counter medications such as selegiline, which is used in the treatment of Parkinson’s disease and major depressive disorder, or famprofazone, a NSAID. On a more positive note, we can conclude that the detection and/or differentiation of co-consumption of illegal D,L-AMP and other drugs was successful. This aspect, in turn, could make it possible to closely monitor misuse among individuals receiving LDX, especially in areas where, from a forensic point of view, concomitant use of other drugs cannot be tolerated, because of its impact in social, criminal, or traffic law.

## Supporting information

S1 File (DOCX)
