# Supplementary material for: Evaluating the reliability of hair analysis in monitoring the compliance of ADHD patients under treatment with Lisdexamphetamine
Source: PLoS One. 2021 Mar 30;16(3):e0248747. doi: 10.1371/journal.pone.0248747 (PMC8009440; doi:10.1371/journal.pone.0248747)
Supplement: S1 File — (DOCX) [file pone.0248747.s001.docx]

**Evaluating the reliability of hair analysis in monitoring the compliance of ADHD patients under treatment with Lisdexamphetamine**

*Marianne Hädener, Wolfgang Weinmann, Dominique Eich, Michael Liebrenz, Thomas Wüthrich, Anna Buadze*

1. **Validation of the chiral LC-MS/MS method for determination of D-AMP and L-AMP in hair samples**

Validation of the chiral LC-MS/MS method for quantification of amphetamine (AMP) enantiomers in hair was performed according to international guidelines on bioanalytical method validation (1, 2). Linearity, lower limit of quantification (LLOQ), intra- and inter-batch accuracy and imprecision, selectivity, recovery, matrix effect, process efficiency, carry-over, and re-injection reproducibility were evaluated.

**1.1 Linearity**

Linearity of each AMP enantiomer was determined with five concentration levels (50, 125, 250, 1250 and 2500 pg/mg) prepared in duplicate on three different days (*n* = 6) and was acceptable if the correlation coefficient (*r*) exceeded 0.99 and calibrators quantified within ± 15% of the target value (± 20% at LLOQ). Calibration curves were generated by calculating the peak area ratio of analyte to internal standard for each analyte concentration (x) and were fit by unweighted linear least-squares regression and linear least-squares regression applying a 1/*x* and 1/*x*^2^ weighting factor, respectively. Inspection of percentage relative errors (3) showed that linear least-squares regression with a 1/*x* weighting factor was the most appropriate calibration model. Calibration curves were linear up to 2500 pg/mg with correlation coefficients *r* > 0.997. Back-calculated values of the calibration standards were within ± 8.2% of the nominal concentrations. The LLOQ was defined as the lowest concentration that can be measured with acceptable accuracy (80 – 120%) and imprecision (≤ 20% CV) and was determined to be 50 pg/mg for both AMP enantiomers, which is in accordance with the recommendations by the Society of Hair Testing (recommended LLOQ for AMP ≤ 200 pg/mg) (4).

**1.2 Accuracy and imprecision**

Accuracy and imprecision were assessed at low, mid, and high quality control (QC) level (150, 500, and 2000 pg/mg per enantiomer) as well as at the LLOQ level. Intra-assay accuracy and imprecision were determined from six replicates per concentration on one day (*n* = 6). Inter-assay accuracy and imprecision were evaluated for six replicates per concentration on three days (*n* total = 18). Accuracy was defined as the mean percentage of all replicates from the nominal concentration and was expected to be 85 – 115% (80 – 120% at LLOQ). Imprecision was expressed as percent coefficient of variation (% CV) of the calculated concentrations and should be < 15% (< 20% at LLOQ). Accuracy and imprecision results are presented in Table S1. Both enantiomers met all acceptance criteria. Accuracy at the four QC levels ranged from 91.3 – 113.7% and imprecision was less than 11.8% CV.

**Table S1.** Intra- and inter-batch accuracy and imprecision data for AMP enantiomers in hair.

**1.3 Method selectivity**

Method selectivity was evaluated by analyzing blank hair samples obtained from six different individuals for absence of signals co-eluting with the analyte peaks. In addition, potential interferences from endogenous compounds were assessed by spiking the different blank hair samples to the LLOQ level. Absence of endogenous interferences was demonstrated if signals in the blank specimens were ≤ 20% of the LLOQ and ≤ 5% of the internal standard response and if the LLOQ samples quantified within ± 20% of the target concentration. No endogenous interferences were present in any of the tested blank hair samples (*n* = 6) and LLOQ quantification was within 14.2% for L-AMP and 12.6% for D-AMP, respectively, thus demonstrating method selectivity.

- 1. **Recovery, matrix effect and process efficiency**

Recovery (RE), matrix effect (ME) and process efficiency (PE) were assessed at low and high QC level using six different hair matrices and three sets of samples as described by Matuszewski et al. (5) (*n* = 6 per QC level in each set). In set *A*, hair samples were spiked prior to extraction. In set *B*, extracted blank hair samples were spiked after extraction, and set *C* consisted of neat QC samples prepared in pure methanol. RE, ME and PE were calculated based on the mean peak areas obtained for each set, according to the following formulas: RE = *A*/*B* x 100; ME = *B*/*C* x 100; and PE = *C*/*A* x 100 = (ME x RE)/100. Results are given in Table S2. Matrix effects for the AMP enantiomers were minor, ranging from 86.4 –100.1%, and were compensated for by their deuterated analogs, which experienced very similar ion suppression/enhancement. Thus, quantification was not adversely affected.

**Table S2.** Recovery, process efficiency, and matrix effect for L- and D-AMP in hair.

- 1. **Carry-over**

Carry-over was investigated in triplicate by injecting a blank sample immediately after the highest calibrator. Carry-over was considered insignificant since any response in the blank specimen was ≤ 20% of the LLOQ and ≤ 5% of the internal standard response.

- 1. **Re-injection reproducibility**

Re-injection reproducibility was demonstrated with six replicates per QC level. These samples were analyzed immediately after extraction and were then re-injected after storage in the autosampler for 7 days at 8 °C and quantified against the re-injected calibration curve. Reproducibility was considered acceptable if re-injected samples quantified within ± 15% of initially measured value. Processed QC samples quantified within ± 9.4% of the first measurement when re-injected after storage in the autosampler for 7 days, thus demonstrating acceptable reproducibility

1. **References**

1. Food and Drug Administration (FDA). Guidance for Industry: Bioanalytical Method Validation. 2001 [Cited 2016 June 30]. Available from: http://academy.gmp-compliance.org/guidemgr/files/4252FNL.PDF

2. European Medicines Agency (EMEA). Guideline on bioanalytical method validation. 2011 [Cited 2016 June 30]. Available from: <http://www.ema.europa.eu/docs/en_GB/document_library/Scientific_guideline/2011/08/WC500109686.pdf>.

3. Almeida AM, Castel-Branco MM, Falcao AC. Linear regression for calibration lines revisited: weighting schemes for bioanalytical methods. Chromatogr B Analyt Technol Biomed Life Sci. 2002;774: 215-22. doi: 10.1016/s1570-0232(02)00244-1.

4. Society of Hair Testing. Recommendations for Hair Testing in Forensic Cases. Forensic Sci Int 2004;145: 83-4. doi: 10.1016/j.forsciint.2004.04.022.

5. Matuszewski BK, Constanzer ML, Chavez-Eng CM. Strategies for the assessment of matrix effect in quantitative bioanalytical methods based on HPLC-MS/MS. Anal Chem. 2003;75: 3019-30. doi: 10.1021/ac020361s.
